# Control in the Hospital by Extensive Clinical rules for Unplanned hospitalizations in older Patients (CHECkUP); study design of a multicentre randomized study

**DOI:** 10.1186/s12877-021-02723-8

**Published:** 2022-01-10

**Authors:** Aimée E. M. J. H. Linkens, Vanja Milosevic, Noémi van Nie, Anne Zwietering, Peter W. de Leeuw, Marjan van den Akker, Jos M. G. A. Schols, Silvia M. A. A. Evers, Carlota Mestres Gonzalvo, Bjorn Winkens, Bob P. A. van de Loo, Louis de Wolf, Lucretia Peeters, Monique de Ree, Bart Spaetgens, Kim P. G. M. Hurkens, Hugo M. van der Kuy

**Affiliations:** 1 Department of Internal Medicine, Division of General Internal Medicine, Section Geriatric Medicine, Maastricht University Medical Centre, PO Box 5800, 6202 AZ Maastricht, The Netherlands; 2 Department of Hospital Pharmacy, Erasmus MC, University Medical Center Rotterdam, 3015 GD Rotterdam, The Netherlands; 3 Clinical Pharmacy, Elkerliek Hospital, Helmond, The Netherlands; 4 Zuyderland Medical Centre, Heerlen, Limburg The Netherlands; 5 Department of Internal Medicine, Geriatric Medicine, Zuyderland Medical Centre, Heerlen, The Netherlands; 6 Department of Internal Medicine, Maastricht University Medical Centre, Maastricht, The Netherlands; 7 Department of Family Medicine, Care and Public Health Research Institute (CAPHRI), Maastricht University, Maastricht, The Netherlands; 8 Institute of General Practice, Goethe University, Frankfurt am Main, Germany; 9 Department of Public Health and Primary Care, KU Leuven, Leuven, Belgium; 10 Department of Health Services Research and Care and Public Health Research Institute (CAPHRI), Maastricht University, Maastricht, The Netherlands; 11 Centre for Economic Evaluation and Machine Learning, Trimbos Institute, Netherlands Institute of Mental Health and Addiction, Utrecht, The Netherlands; 12 Clinical Pharmacy and Toxicology, Maastricht University Medical Centre+, Maastricht, Limburg The Netherlands; 13 Department of Methodology and Statistics, Care and Public Health Research Institute (CAPHRI), Maastricht University, Maastricht, The Netherlands; 14 Digitalis Rx BV, Amsterdam, The Netherlands; 15 General Practitioner, Stein, The Netherlands; 16 General Pharmacist, Heerlen, The Netherlands; 17 General Pharmacist, Susteren, The Netherlands; 18 Department of Cardiovascular Research Institute Maastricht (CARIM), Maastricht University, Maastricht, The Netherlands

**Keywords:** Older patients, Polypharmacy, Readmissions, Medication optimisation, Clinical decision support system

## Abstract

**Background:**

Due to ageing of the population the incidence of multimorbidity and polypharmacy is rising. Polypharmacy is a risk factor for medication-related (re)admission and therefore places a significant burden on the healthcare system. The reported incidence of medication-related (re)admissions varies widely due to the lack of a clear definition. Some medications are known to increase the risk for medication-related admission and are therefore published in the triggerlist of the Dutch guideline for Polypharmacy in older patients. Different interventions to support medication optimization have been studied to reduce medication-related (re)admissions. However, the optimal template of medication optimization is still unknown, which contributes to the large heterogeneity of their effect on hospital readmissions. Therefore, we implemented a clinical decision support system (CDSS) to optimize medication lists and investigate whether continuous use of a CDSS reduces the number of hospital readmissions in older patients, who previously have had an unplanned probably medication-related hospitalization.

**Methods:**

The CHECkUP study is a multicentre randomized study in older (≥60 years) patients with an unplanned hospitalization, polypharmacy (≥5 medications) and using at least two medications from the triggerlist, from Zuyderland Medical Centre and Maastricht University Medical Centre+ in the Netherlands. Patients will be randomized. The intervention consists of continuous (weekly) use of a CDSS, which generates a Medication Optimization Profile, which will be sent to the patient’s general practitioner and pharmacist. The control group will receive standard care. The primary outcome is hospital readmission within 1 year after study inclusion. Secondary outcomes are one-year mortality, number of emergency department visits, nursing home admissions, time to hospital readmissions and we will evaluate the quality of life and socio-economic status.

**Discussion:**

This study is expected to add evidence on the knowledge of medication optimization and whether use of a continuous CDSS ameliorates the risk of adverse outcomes in older patients, already at an increased risk of medication-related (re)admission. To our knowledge, this is the first large study, providing one-year follow-up data and reporting not only on quality of care indicators, but also on quality-of-life.

**Trial registration:**

The trial was registered in the Netherlands Trial Register on October 14, 2018, identifier: NL7449 (NTR7691). https://www.trialregister.nl/trial/7449.

**Supplementary Information:**

The online version contains supplementary material available at 10.1186/s12877-021-02723-8.

## Background

The population is ageing, leading to an increased incidence of multimorbidity and related polypharmacy [1]. Polypharmacy is a well-known risk factor for hospital (re)admissions, which can have detrimental effects on older patients and therefore are considered an important measure of quality of care [2]. As such, it is not surprising that a significant number of these hospital readmissions is directly medication-related and that medication-related hospital (re)admissions occur more frequently in older individuals [3, 4].

The incidence of both medication-related hospital admissions and readmissions varies widely, ranging from 0.5 to 19.3 and 0.09% to 64.0%, respectively [5]. Several explanations might be given for this wide range. First, there is lack of a clear definition of “medication-related hospital admission” and “medication-related hospital readmission”. Most definitions are based on the assumption that (re)admissions are directly related to problems around pharmacotherapy and are defined as (I) drug-related problems, such as drug-drug interactions, inappropriate drug use, sub- and supra-therapeutic dosage, and adverse drug reactions [3, 6]. Second, another explanation for the wide range in incidence of medication-related hospital (re)admissions might be the difference in time-at-risk of adverse outcome, i.e. the time between discharge after the first hospital admission and subsequent readmission in different studies. The follow-up time of these studies ranges from 30 days to 3 years and it is self-evident that the percentages of readmissions rise substantially when the follow-up time increases [6–12]. Third, medication-related (re)admissions are probably under recognized, especially in older patients who often tend to have an atypical presentation of illness.

While there is ongoing discussion and a clear definition about medication-related hospital (re)admissions is lacking, the Dutch multidisciplinary guideline for polypharmacy in older patients published the (so-called) triggerlist with clinical events (triggers) and often involved medications that are known to be associated with an increased risk of medication-related admissions [13]. As such, this list could serve as a guide whether to call a hospital (re)admission medication-related. Table 1 shows the triggerlist, which is compiled based on data from the HARM-, IPCI- and QUADRAT studies [14–17].

**Table 1:** Triggerlist from the Dutch guideline “Polypharmacy in the older patient” [13]

Trigger (adverse clinical event)	Often involved medication
Fracture / fall	Psychotropic medication (falls)/ corticosteroids / antihypertensive drugs
Collapse / hypotension / dizziness	Cardiac medication (antihypertensive drugs and antiarrhythmics)/ psychotropic medication
Bleeding (GI tract)/ supratherapeutic INR	Anticoagulants Antiplatelet drugs NSAID
Electrolyte imbalance / dehydration	Diuretics, ACEi, AII-blocker, NSAID, antidepressants
Renal insufficiency	ACEi, AII-blocker, NSAID
Hypo- or hyperglycaemia	Insulin/oral antidiabetics, Corticosteroids
Heart failure	NSAID
Obstipation / ileus	Opioids / calcium blockers
Vomiting / diarrhea	Antibiotics
Delirium / confusion / drowsiness	Psychotropic medication / cardiac medication / medication for micturition complaints / benzodiazepines

In order to reduce medication-related hospital (re)admissions, several interventions that involve medication review have been investigated. Although a recent systematic review showed that an isolated medication review had no effect on readmission rates, multiple studies claim the opposite by showing involvement of a pharmacist does lead to a reduction in readmission rates [18–21]. This discrepancy might be explained by the fact that medication reviews often are a part of more comprehensive interventions [22] and also that pharmacists do not just perform isolated medication reviews, but often (implicitly) combine it to a multifaceted program that includes medication reconciliation, patient counseling and adequate follow-up [23]. Nevertheless, these programs performed during admission are very time consuming, relatively expensive and the quality may vary considerably between pharmacists [21]. To overcome these problems a clinical decision support system (CDSS) that monitors medication and patient characteristics continuously and sends recommendations to general practitioners (GPs) and pharmacists after detecting a medication-related problem, could be used [24]. Consequently, possible medication-related problems will be detected immediately in contrast to manual medication reviews that are usually performed only once or twice a year. Currently available research on the continuous use of a CDSS mainly focuses on the inpatient (hospital and nursing home) setting [24–26]. As such, there exists a critical knowledge gap in the outpatient setting that needs to be addressed.

In view of the considerations above, the aim of this study is to investigate whether the continuous use of a CDSS decreases the number of hospital readmissions in older patients who previously have had an unplanned probably medication-related hospitalization according to the triggerlist from The Dutch multidisciplinary guideline for polypharmacy in older patients [13].

## Methods/design

### Study design and setting

The “Control in the Hospital by Extensive Clinical rules for Unplanned hospitalizations in older Patients” (CHECkUP) is a multicentre, prospective and randomized study. This study will be embedded in two hospitals namely Zuyderland Medical Centre (MC) (location Sittard-Geleen and location Heerlen) and Maastricht University Medical Centre + (MUMC+), The Netherlands. Zuyderland MC is a large teaching hospital and MUMC+ is an academic hospital. The patients will be randomized by block randomizations with a size of two. This study is blinded for patients as well as for the participating GPs and pharmacists.

### Study population

All patients aged 60 years and older with an unplanned hospital admission are eligible for inclusion if they meet the inclusion criteria. The inclusion criteria are polypharmacy (defined as using ≥5 medications chronically), using at least two medications from the triggerlist and the ability to give informed consent.

Patients with a life expectancy of less than 3 months (assessed by the involved practitioners); patients with an intentional auto-intoxication; and patients treated with cytostatic will be excluded.

### Outcomes

#### Primary outcome

The primary outcome of this study is hospital readmission within 1 year after study inclusion.

#### Secondary outcomes

The secondary outcomes of this study are (I) mortality within 1 year after study inclusion; (II) the number of emergency department visits; (III) the number of nursing home admissions; (IV) time to hospital readmission; and (V) the number of hospital readmissions after 30 and 180 days. Next, we will analyze whether the readmission is (probably) medication-related. Whether a hospital readmission is medication-related will be defined afterwards, using the triggerlist. The information for secondary outcomes will be obtained from the electronic prescription system and electronic patient record. Furthermore, quality of life (QoL) and costs measured from a societal perspective will be assessed at baseline (hospital discharge) and after 3, 6 and/or 12 months (see questionnaires).

### Study procedures

Three times a week the electronic prescription system is used to select the patients of 60 years and older admitted to the hospital with polypharmacy and at least two medications from the triggerlist. A research nurse will visit the patient at the ward and assess whether the patient is eligible for inclusion. The patients will receive written information about the study and after 2 days the research nurse will visit them again. Then they have to indicate whether they are willing to participate by signing informed consent. Inclusion already started in April 2019 and will finish in August 2022.

**Fig. 1:**
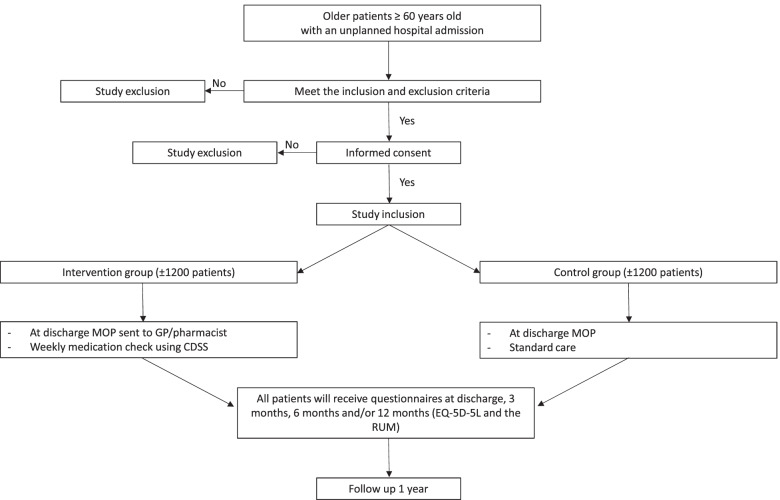
Study design and randomization procedure of CHECkUP. Legend: MOP: Medication Optimisation Profile; GP: General practitioner; CDSS: clinical decision support system; EQ-5D-5L: EuroQol-5D-5L; RUM: Resource Use Measurement

After inclusion, patients will be randomized into the intervention or the control group by using a digital randomization system with block randomization. The randomization is blinded for the patients, the GP and the pharmacist. Figure 1 illustrates the study design and randomization procedure.

### CDSS

**Fig. 2:**
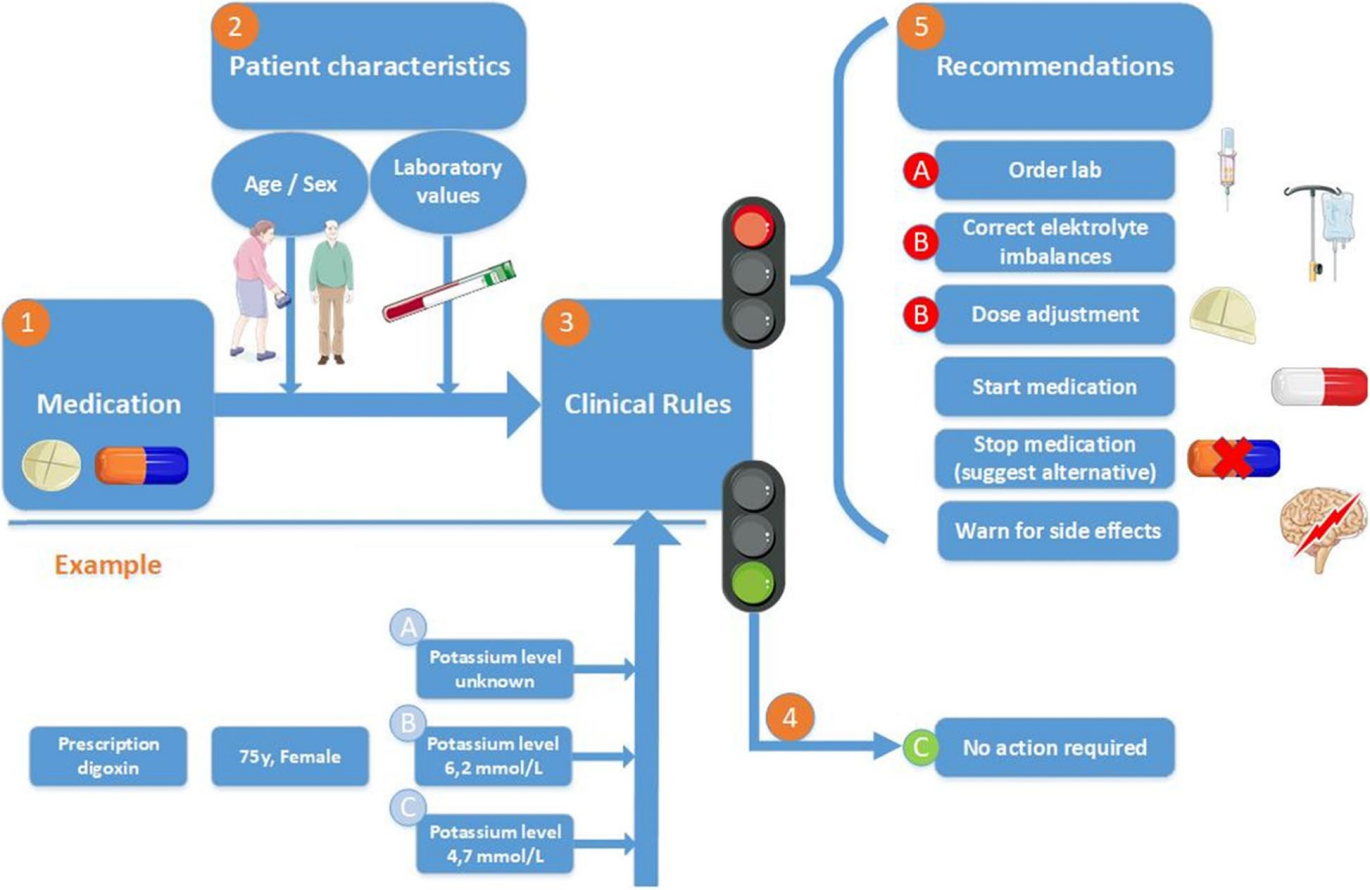
Schematic overview of the CDSS and example. Legend: When running the CDSS, the patient’s medication list (1) is combined with his/her characteristics (2), such as age, sex and laboratory values (renal function, potassium level etc.). Next, these data are run through the 225 different clinical rules (3). When no clinical rules apply, a green signal is given (4) and no further actions are required (**C**). When clinical rules do apply a red signal is given and clinical recommendations (5) will be sent to the GP and/or pharmacist. The figure also shows an example of a 75 year-old female that is prescribed digoxin. The clinical rule about ‘potassium and digoxin’ is applied and different scenario’s in which the potassium level is unknown (**A**), 6.2 mmol/L (**B**) or 4.7 mmol/L (**C**) lead to different clinical recommendations with the recommendation to order lab (**A**), correct electrolyte imbalances or dose adjustment (**B**) or no action is required (**C**), respectively. This figure was created using Servier Medical Art templates, which are licensed under a Creative Commons Attribution 3.0 Unported License; https://smart.servier.com

In the present study we use a CDSS (we use the Clinical Rule Reporter, developed by Digitalis) to optimise medication on a continuous basis, ensuring that new medication interactions or problems, e.g. related to comorbidity, laboratory data (renal function) are quickly identified. This software has been validated and is currently used in multiple settings i.e. hospital and nursing homes [24–26]. The CDSS analyses the pharmacotherapy of patients using data regarding the patient’s medication, patient characteristics such as age, sex and laboratory values, and different guidelines/criteria specific for medication assessment, such as the START/STOPP criteria [24, 25, 27]. Combined with the patient’s medication list and characteristics, these different guidelines and criteria are summarized in 151 different clinical rules (see Additional file 1, Table S1). These clinical rules aim to optimize the medication list and gives clinically relevant recommendations, such as lab orders, dose adjustment, stop medication. Then, the different recommendations per patients are summarized into a Medication Optimization Profile (MOP). Figure 2 shows a schematic overview of the CDSS and an example of how different characteristics can trigger different recommendations.

### Participation GPs and pharmacist

During the 1 year study period, the patient’s own GP and pharmacist receive the MOPs, to optimize pharmacotherapy. All GPs and pharmacists in the region were approached to voluntarily participate in the study. Only patients of GPs and pharmacists who had indicated to take part in the study will be able to participate.

### Intervention group

Patients included in the intervention group will undergo continuous medication checks using the CDSS once the patient is discharged. At discharge, the GP and pharmacist will receive their patient’s MOP. From then on the MOPs will be sent on a weekly basis for the period of 1 year. The GPs and the pharmacists can access this MOP and make necessary changes to the medication when appropriate. When they decide not to follow the recommendation made by the CDSS they are asked to indicate a reason.

### Control group

The control group will receive standard care. The GPs and the pharmacists are not informed of which patients are participating as control. Therefore the GPs and pharmacists are blinded for this part of the study. The CDSS will also generate a MOP at discharge for patients in the control group. However this will not be sent to the patient’s GP and pharmacist and is only generated for analysis at the end of the study.

### Questionnaires

All patients (both intervention and control group) will be sent standardized questionnaires about the quality of life (EuroQol 5D-5L (EQ-5D-5L)) and costs from a societal perspective (Resource Use Measurement (RUM)) at hospital discharge, and after 3, 6 and/or 12 months after inclusion [28, 29]. The research nurse will determine whether the included patient will receive the second RUM questionnaire at 3, 6 or 12 months after inclusion, in order to make sure that the groups are equal. The patients will receive the questionnaires via email and receive an automatically generated reminder after 1 week. The questionnaire must be completed within 1 month.

### Sample size calculation

Based on a pilot-study the readmission rate for this selected group is estimated to be 20% [30]. The aim of this study is to reduce the readmission rate from 20 to 15%. To demonstrate this reduction (power 80%; significance level 5%; dropout 20%) at least 1130 evaluable patients are necessary per group. The target population is 2400 patients. The study will include 1200 patients in the intervention group and 1200 patients in the control group divided over the two hospitals with a minimum of 600 patients per location (300 per group).

### Data analysis / statistical analysis

The effect evaluation will be analyzed according to the intention to treat principle. The difference in primary and secondary outcome variables between the intervention and the control group will be assessed using mixed effect models to account for the clustering of patients within physician and/or repeated measurements. A logit link function will be used for the binary outcomes and an identify link for numerical outcomes. A likelihood-based approach will be applied to account for missing outcomes variables, assuming missingness at random (MAR). The stratification variable (hospital location), variables related to missing data/drop outs (to ensure MAR) and variables related to the outcome such as age and sex, will be included in the fixed part of the models.

### Economic evaluation/ cost analysis

The economic evaluation will be performed according to the Dutch guidelines of the national health care institute [31]. As mentioned earlier, the study will include 1200 patients in the intervention group and 1200 patients in the control group. As it is impossible to follow each patient, during 1 year follow-up we will use intermittent data collection instead of continuous data collection, because results showed that the best estimations of annual impact can be obtained by random cohort data collection, using 3 random cohorts, enduring that at least a third of the participants will be measured at each measured point [31]. Intermittent measurement combined with individual mean (IM_ imputation) will be used to calculate the annual costs per Quality Adjusted Life Years (QALYs). This means that at each measurement point 400 patients per group will complete the RUM instrument for the costs and EQ-5D-5L for the QALYs during every three – 6 months after inclusion.

## Discussion

This study is expected to add evidence on the knowledge of medication optimization and whether continuous use of a CDSS ameliorates the risk of hospital readmission and other adverse outcomes in older patients who have already had an unplanned hospitalization.

Hospital (re)admissions place a significant burden on the healthcare system, with impact on quality of life from the patient perspective and being an important cost driver from the societal perspective. In earlier studies, different interventions have been investigated to reduce readmissions, but the results were inconclusive due to large heterogeneity in study designs and therefore their effect on hospital readmissions. As such, the optimal template of medication optimization is still unknown. This is the first study that includes patients with a high risk of having a medication-related admission based on the triggerlist. We deliberately chose to include all readmissions as primary outcome and not specifically medication-related readmissions because a clear definition is lacking. By using a suboptimal definition it is likely we would miss readmissions that later turn out to be medication-related.

To our knowledge, this is the first large randomized blinded study providing one-year follow-up data and reporting not only on quality of care indicators (readmissions), but also on quality-of-life and costs. This contrasts to other studies in the field, which usually have a follow up of 30 or 60 days at most. This is an important strength of our study, while we believe that in this population healthcare status and medication prescriptions alter frequently and a follow up of only 30 or 60 days is too short to identify all possible consequences and the time to the occurrence of adverse outcome might vary considerably. Another strength of this study is that it will be conducted in the outpatient setting and directly in daily clinical practice and therefore improves the possibility to implement the CDSS in the shortest possible notice. The inclusion of patients has already started in April 2019, but the patient inclusion was slower than expected due to different causes. The participation of GPs and pharmacists was lower than expected, we experienced several IT problems (not related to CDSS itself) that affected inclusion and from March 2020 we had to deal with the COVID-19 pandemic. As such, during many months the inclusion was discontinued.

In conclusion, we strongly believe that the continuous use of a CDSS reduces the number of hospital readmissions in older patients already at an increased risk of medication-related hospital admission. It is of vital importance to determine the optimal template of medication optimization and further improve this essential process to eventually achieve high-quality and cost-effective care, especially in older patients with polypharmacy.

## Supplementary Information

**Additional file 1: Table S1.** Overview of the clinical rules.

## Data Availability

A de-identified dataset may be made available from the corresponding author on reasonable request once the study is completed.

## Abbreviations

CDSS: Clinical decision support system
CHECkUP: Control in the Hospital by Extensive Clinical rules for Unplanned hospitalizations in older Patients
EuroQol 5D-5L: EQ-5D-5L
GP: General practitioner
MAR: Missingness at random
MC: Medical Centre
MOP: Medication Optimization Profile
MUMC+: Maastricht University Medical Centre +
QALYs: Quality Adjusted Life Years
QoL: Quality of life
RUM: Resource Use Measurement
