# Supplementary material for: Control in the Hospital by Extensive Clinical rules for Unplanned hospitalizations in older Patients (CHECkUP); study design of a multicentre randomized study
Source: BMC Geriatr. 2022 Jan 10;22:36. doi: 10.1186/s12877-021-02723-8 (PMC8744034; doi:10.1186/s12877-021-02723-8)
Supplement: Supplementary file 1 — Additional file 1: Table S1. Overview of the clinical rules. [file 12877_2021_2723_MOESM1_ESM.docx]

Additional File 1

Table S1. Overview of the clinical rules

|  | **Title of the rule** |
| --- | --- |
| 1 | Check whether Benzodiazepine deprescribing is possible |
| 2 | Check whether Brotizolam deprescribing is possible |
| 3 | Check whether Flunitrazepam deprescribing is possible |
| 4 | Check whether Flurazepam deprescribing is possible |
| 5 | Check whether Loprazolam deprescribing is possible |
| 6 | Check whether Lorazepam deprescribing is possible |
| 7 | Check whether Lormetazepam deprescribing is possible |
| 8 | Check whether Midazolam deprescribing is possible |
| 9 | Check whether Nitrazepam deprescribing is possible |
| 10 | Check whether Oxazepam deprescribing is possible |
| 11 | Check whether Temazepam deprescribing is possible |
| 12 | Check whether Zolpidem deprescribing is possible |
| 13 | Check whether Zopiclon deprescribing is possible |
| 14 | Gastric protection (version 2) |
| 15 | Treatment with antihypertensive medications: Angiotensin-converting enzyme (ACE) inhibitors and angiotensin II receptor blockers (ARBs) |
| 16 | Low-density lipoprotein (LDL) optimalisation |
| 17 | MDRD & ACE for older patient |
| 18 | MDRD or CKD-EPI and ACR are required |
| 19 | Carbamazepine/Oxcarbamazepine + hyponatraemia |
| 20 | Potassium levels |
| 21 | Potassium levels + digoxin |
| 22 | selective serotonin reuptake inhibitor (SSRI's) with significant hyponatremia |
| 23 | Thiazides and hyponatraemia |
| 24 | Unknown potassium serum level |
| 25 | MDRD required |
| 26 | Renal dysfunction + Aciclovir - Oral |
| 27 | Renal dysfunction + Acipimox |
| 28 | Renal dysfunction + Alendronic acid |
| 29 | Renal dysfunction + Amantadine |
| 30 | Renal dysfunction + Amoxicillin |
| 31 | Renal dysfunction + Apixaban |
| 32 | Renal dysfunction + Barnidipine |
| 33 | Renal dysfunction + Benzylpenicillin |
| 34 | Renal dysfunction + Carbasalate calcium (analgesic) |
| 35 | Renal dysfunction + Cefalexin |
| 36 | Renal dysfunction + Cefazolin |
| 37 | Renal dysfunction + Cefotaxime |
| 38 | Renal dysfunction + Ceftazidime |
| 39 | Renal dysfunction + Cefuroxime |
| 40 | Renal dysfunction + Cetirizine |
| 41 | Renal dysfunction + Chloroquine |
| 42 | Renal dysfunction + Chlortalidone |
| 43 | Renal dysfunction + Cimetidine |
| 44 | Renal dysfunction + Ciprofloxacin - IV |
| 45 | Renal dysfunction + Ciprofloxacin - Oral |
| 46 | Renal dysfunction + Co-amoxiclav |
| 47 | Renal dysfunction + Colchicine |
| 48 | Renal dysfunction + Dabigatran |
| 49 | Renal dysfunction + Dalteparine |
| 50 | Renal dysfunction + Danaparoide |
| 51 | Renal dysfunction + Daptomycine |
| 52 | Renal dysfunction + Deferasirox |
| 53 | Renal dysfunction + Edoxaban |
| 54 | Renal dysfunction + Entecavir |
| 55 | Renal dysfunction + Ertapenem |
| 56 | Renal dysfunction + Fluconazole |
| 57 | Renal dysfunction + Ganciclovir |
| 58 | Renal dysfunction + Hydroxychloroquine |
| 59 | Renal dysfunction + Imipenem/cilastatin |
| 60 | Renal dysfunction + Lacosamide |
| 61 | Renal dysfunction + Levetiracetam |
| 62 | Renal dysfunction + Levocetirizine |
| 63 | Renal dysfunction + Levofloxacin |
| 64 | Renal dysfunction + Lithium |
| 65 | Renal dysfunction + Memantine |
| 66 | Renal dysfunction + Meropenem |
| 67 | Renal dysfunction + Metformine |
| 68 | Renal dysfunction + Midazolam |
| 69 | Renal dysfunction + Nitrofurantoin |
| 70 | Renal dysfunction + Norfloxacin |
| 71 | Renal dysfunction + Ofloxacin |
| 72 | Renal dysfunction + Oseltamivir |
| 73 | Renal dysfunction + Paliperidone |
| 74 | Renal dysfunction + Parathyroid hormone |
| 75 | Renal dysfunction + Pergolide |
| 76 | Renal dysfunction + Piracetam |
| 77 | Renal dysfunction + Pramipexole |
| 78 | Renal dysfunction + Probenecid |
| 79 | Renal dysfunction + Proguanil |
| 80 | Renal dysfunction + Quinine |
| 81 | Renal dysfunction + Risperidone |
| 82 | Renal dysfunction + Rivaroxaban |
| 83 | Renal dysfunction + Rosuvastatin |
| 84 | Renal dysfunction + Solifenacin |
| 85 | Renal dysfunction + Sotalol (1) |
| 86 | Renal dysfunction + Sotalol (2) |
| 87 | Renal dysfunction + Sucralfate |
| 88 | Renal dysfunction + Sulfadiazine |
| 89 | Renal dysfunction + Tazocin/Piperacillin |
| 90 | Renal dysfunction + Teicoplanin |
| 91 | Renal dysfunction + Terbinafine |
| 92 | Renal dysfunction + Tetracycline |
| 93 | Renal dysfunction + Tranexamic acid |
| 94 | Renal dysfunction + Valaciclovir |
| 95 | Renal dysfunction + Varenicline |
| 96 | Renal dysfunction + Venlafaxine |
| 97 | Acetosal and acenocoumarol or fenprocoumon |
| 98 | Bisphosphonates and calcium, and vitamin D supplementation |
| 99 | Bisphosphonates, Calcium and Vitamin D supplementation |
| 100 | Chronic use of laxatives |
| 101 | Concomitant use of an ACE-inhibitor with codeine |
| 102 | Diclofenac, celecoxib and etoricoxib should not be used in patients with angina pectoris or ischemic heart disease |
| 103 | Diclofenac, celecoxib and etoricoxib should not be used in patients with stroke in the anamnesis |
| 104 | Dipyridamol usage and no antihypertensive medication |
| 105 | Gastric protection |
| 106 | Nitrate and no beta blocker |
| 107 | Use of dipyridamol without acetosal |
| 108 | Use of LMWH and acenocoumarol for more than 5 days |
| 109 | Anticholinergic antispasmodic drugs + chronic constipation |
| 110 | Anticholinergic antispasmodic drugs + chronic constipation |
| 111 | Anticholinergics to treat extrapyramidal side effects of neuroleptic medications |
| 112 | Aspirin, clopidogrel or dipyridamole + concurrent bleeding disorder |
| 113 | Beta blockers with diabetes mellitus(DM) and hypoglycaemic episode a month |
| 114 | Bladder antimuscarinic drugs with constipation |
| 115 | Bladder antimuscarinic drugs with constipation |
| 116 | Bladder antimuscarinic drugs with dementia |
| 117 | Bladder antimuscarinic drugs with dementia |
| 118 | Bladder antimuscarinic drugs with glaucoma |
| 119 | Bladder antimuscarinic drugs with glaucoma |
| 120 | Bladder antimuscarinic drugs with prostatism |
| 121 | Bladder antimuscarinic drugs with prostatism |
| 122 | Calcium channel blockers with chronic constipation (1) |
| 123 | Calcium channel blockers with chronic constipation (2) |
| 124 | Estrogens with history of breast cancer or venous thromboembolism |
| 125 | Glibenclamide or Chlorpropamide with DM type 2 |
| 126 | Glibenclamide or Chlorpropamide with DM type 2 |
| 127 | Long term neuroleptics with parkinsonism |
| 128 | Long term neuroleptics with parkinsonism |
| 129 | Long-acting Benzodiazepines or long-acting metabolites |
| 130 | Nebulized ipratropium and/or beta2 adrenergics with glaucoma |
| 131 | Nebulized ipratropium and/or beta2 adrenergics with glaucoma |
| 132 | Non-cardioselective beta-blocker with Chronic obstructive pulmonary disease (COPD) |
| 133 | Non-cardioselective beta-blocker with COPD |
| 134 | Phenothiazines in patients with epilepsy |
| 135 | Phenothiazines in patients with epilepsy |
| 136 | Prochlorperazine or metoclopramide with Parkinsonism |
| 137 | Prochlorperazine or metoclopramide with Parkinsonism |
| 138 | Prolonged use of 1st generation antihistamines |
| 139 | Theophylline as monotherapy for COPD |
| 140 | Thiazide diuretic with a history of gout (1) |
| 141 | Thiazide diuretic with a history of gout (2) |
| 142 | Tricyclic antidepressants with cardiac conductive abnormalities (1) |
| 143 | Tricyclic antidepressants with cardiac conductive abnormalities (2) |
| 144 | Tricyclic antidepressants with constipation (1) |
| 145 | Tricyclic antidepressants with constipation (2) |
| 146 | Tricyclic antidepressants with dementia (1) |
| 147 | Tricyclic antidepressants with dementia (2) |
| 148 | Tricyclic antidepressants with glaucoma (1) |
| 149 | Tricyclic antidepressants with glaucoma (2) |
| 150 | Tricyclic antidepressants with prostatism or prior history of urinary retention (1) |
| 151 | Tricyclic antidepressants with prostatism or prior history of urinary retention (2) |
